# Neointimal dissection – a rare complication to endovascular treatment in grafts and stent grafts

**DOI:** 10.1186/s42155-023-00401-x

**Published:** 2023-10-23

**Authors:** Anne Sofie F. Larsen, Shakil Aslam, Lars Olaf Holmen

**Affiliations:** 1 Radiology Department, Ostfold Hospital Trust, PB 300, Kalnesveien 300, 1714 Gralum, Norway; 2 Surgery Department, Ostfold Hospital Trust, Gralum, Norway

**Keywords:** Neointima, Fibrin sheet, Dissection, Graft, Stent graft, Endovascular treatment

## Abstract

**Background:**

Neointima formation and hyperplasia in vascular grafts may lead to graft complications threatening the patency of the vascular reconstruction. A rare complication to endovascular treatment of grafts and stent grafts is dissection inside the graft.

**Case report:**

We present here a case of a 69-year-old female with acute occlusion of the limb of an aorto-bifemoral graft for the third time, 16 years after the primary operation. As at the first two occasions, catheter-based intra-arterial thrombolysis was performed, but with residual stenosis inside the graft. During stent placement, dissection of the neointima or fibrin sheet occluded the inflow to the stent. The complication was resolved with placement of kissing stents.

**Conclusions:**

It is important to recognize iatrogenic neointima dissection inside graft and stent grafts, as continued thrombolysis will not solve this, but increase the risk of hemorrhagic complications.

## Background

Neointima formation and hyperplasia in vascular grafts may lead to graft complications threatening the patency of the vascular reconstruction [1]. Endovascular treatment of anastomotic stenosis is an established treatment option, as well as thrombolytic treatment in late occlusions. Endovascular treatment of neointimal anastomotic lesions may differ from treatment of native vessels, with resistance to angioplasty and shorter patency. A rare complication to endovascular treatment of grafts and stent grafts is dissection inside the graft. It is important to recognize this entity, as dissections of neointima or chronic fibrin sheets may behave differently from intimal dissections of a native vessel.

## Case report

A 69-year-old female patient with a long vascular history and atrial flutter, presented with acute critical limb ischemia of the left extremity after stopping her anticoagulation (apixaban).

She had received an aortobifemoral graft at the age of 53, and had two prior episodes with acute occlusion of the right limb of the graft, 10 and 14 years after the primary operation. Both occlusions was resolved with catheter-directed thrombolysis, the last time with application of a stent in the proximal part of the right limb of the graft.

CTA confirmed occlusion of the left leg of the aortic-bifemoral bypass, and new short occlusions of two calf arteries. There was also wall thrombus in the native aorta above the graft, as well as in the main body of the graft.

After discussion with the vascular surgeon, we started catheter-based intraluminal tPA-administration via a crossover hook-shaped catheter, with the intention to aspirate residual emboli from the calf the next day if needed.

**Fig. 1:**
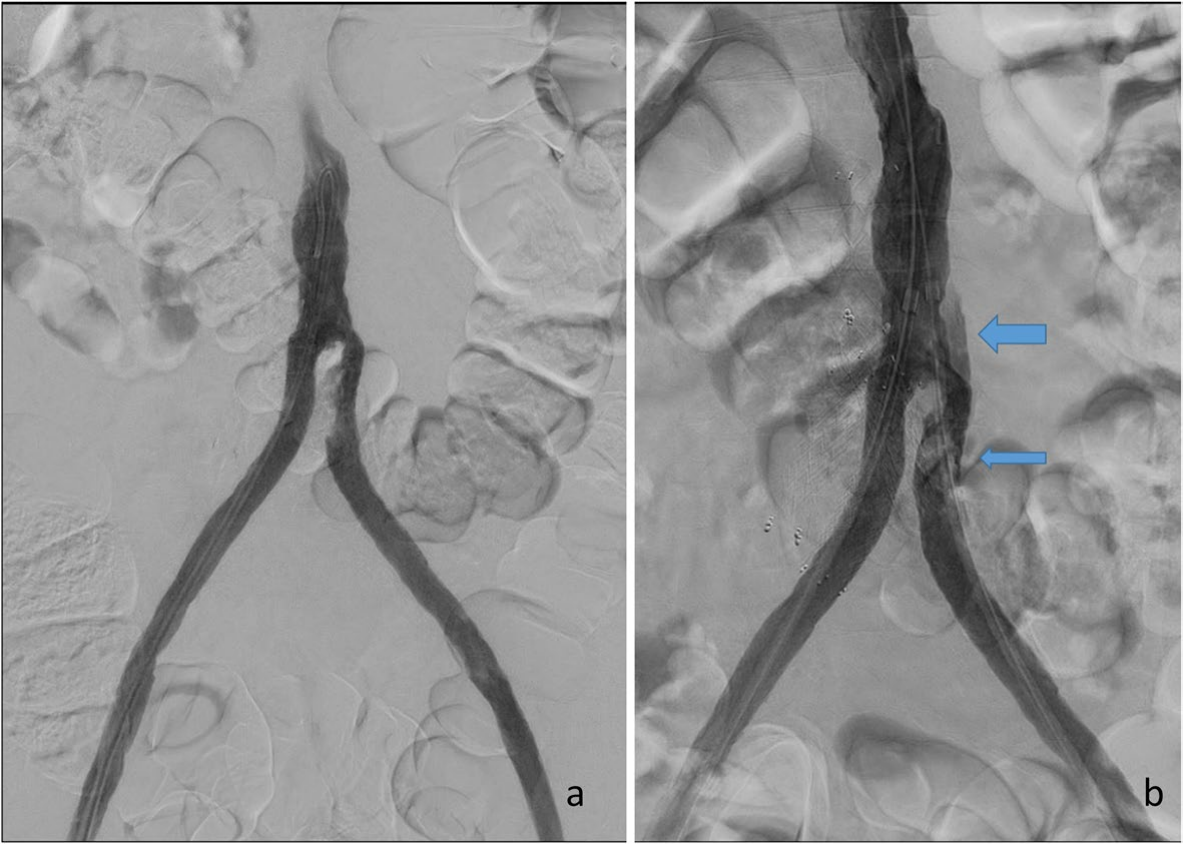
After 12 h of thrombolysis, there is residual stenosis in the proximal part of the left aortofemoral limb (**a**). After placement of a sheet in the left groin, there is some thrombus formation or loosening (arrow), as well as a dissection of thrombus or neointima (broad arrow) (**b**)

**Fig. 2:**
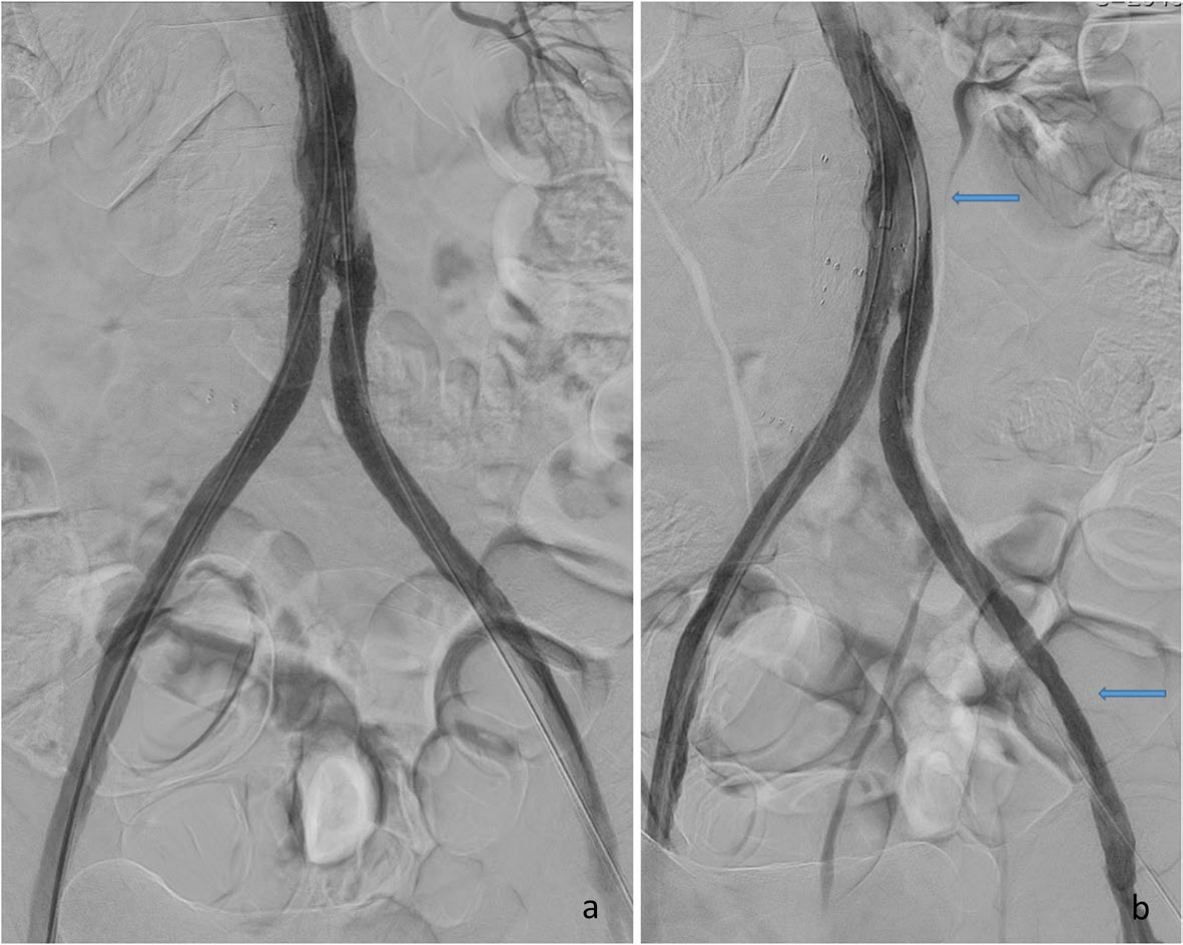
After placement of kissing stents, there are some irregularities proximal to the left stent (**a**). The stents were extended proximally, but control angiogram show dissection at two levels (arrows) (**b**)

**Fig. 3:**
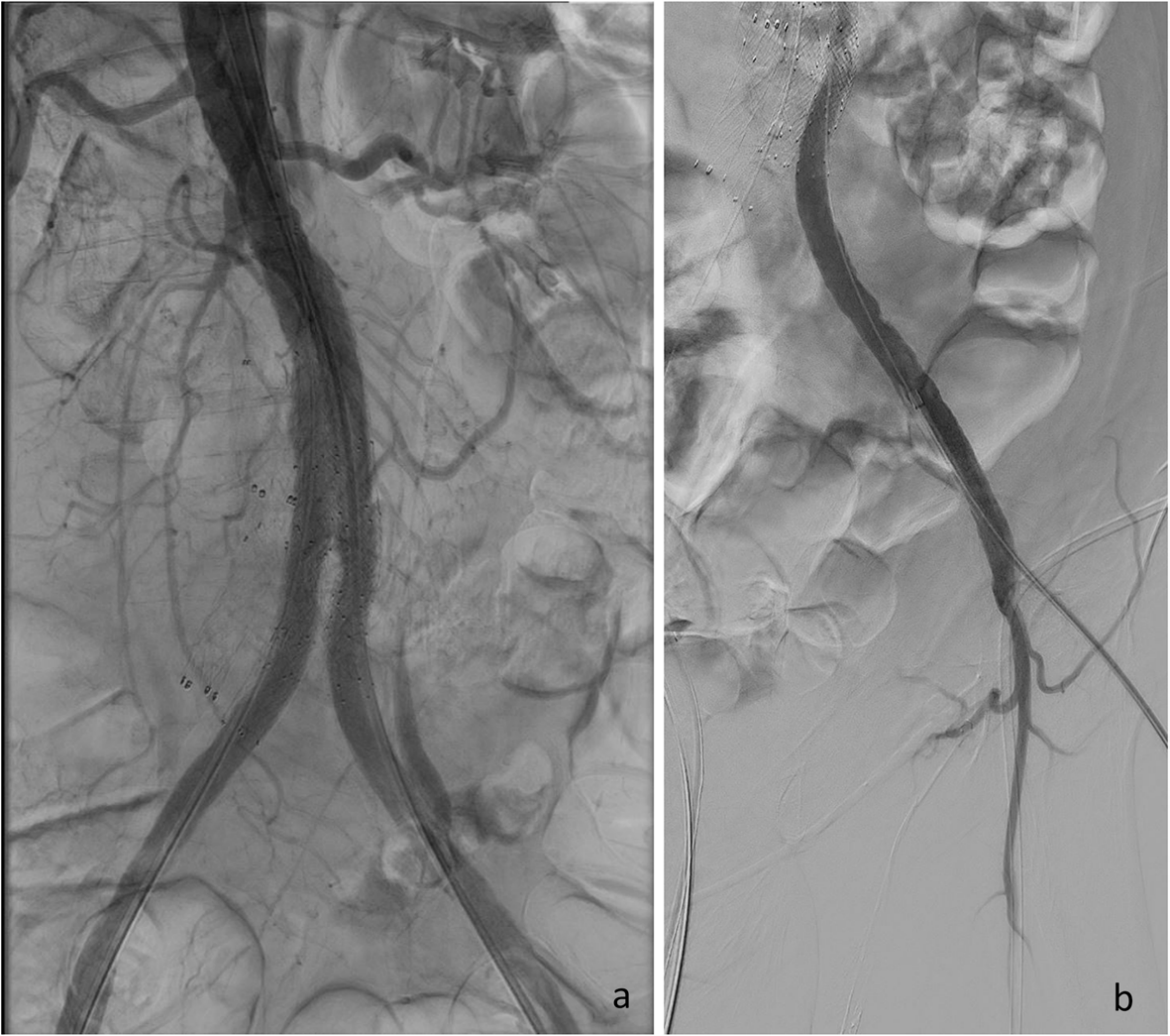
Final angiogram after kissing stents all the way up to the proximal anastomosis of the graft (**a**), and after repositioning of the sheet to the true lumen (**b**)

**Fig. 4:**
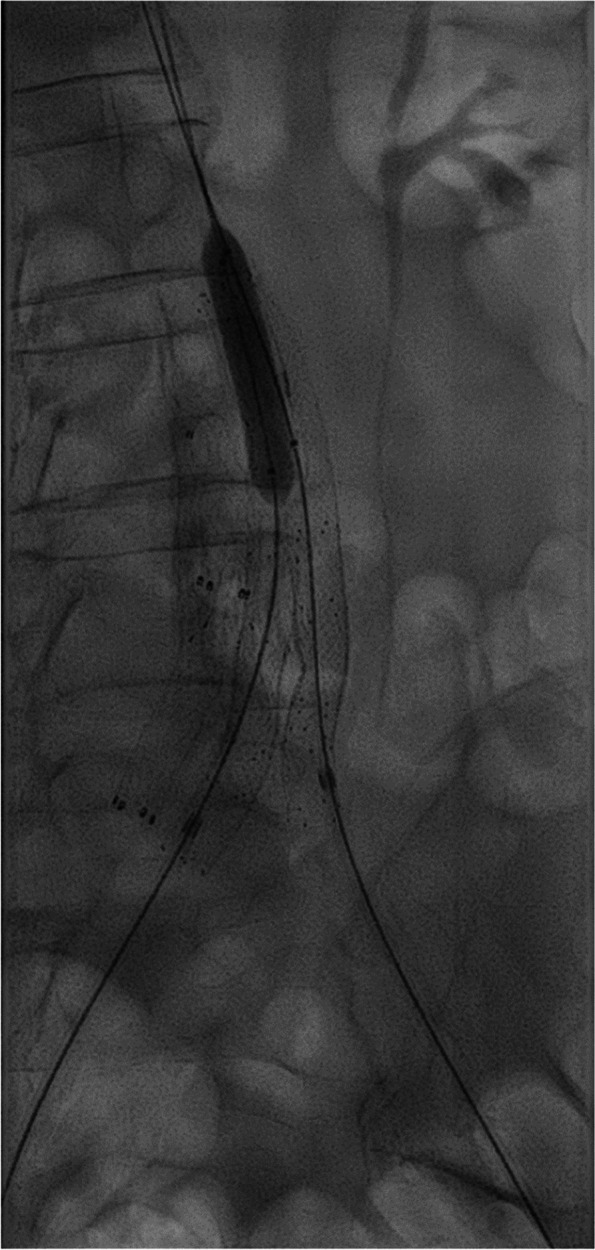
The kissing stents was been prolonged up thru the main body of the graft, and balloon inflated. Occluded stents from prior treatment in the background

After 12 h, the thrombus was dissolved. There was significant residual stenosis due to neointimal hyperplasia or wall adherent thrombus in the proximal limb, and the left groin was punctured to apply kissing stents at the bifurcation of the graft (Fig. 1a and b). Control angiograms after stent placement showed restricted flow through the left limb, with correct placement of the wire in the aorta above the graft anastomosis (Fig. 2a and b). The findings of the angiograms were consistent with neointimal dissection at two levels. At the proximal level, the stent was deployed inside the false lumen without inflow, but with the distal end inside the true lumen. The proximal neointimal dissection could only be resolved with prolonging the kissing stents all the way to the proximal graft anastomosis (Figs. 3 a, b and 4). The distal dissection was not flow limiting after the wire was moved to the correct lumen via buddy wire technique, and this dissection was left untreated.

Emboli to the left calf were aspirated via a new antegrade puncture in the left groin, but completion angiograms showed some residual thrombi and a chronic occlusion of the dorsal pedal artery.

The patient is back to her habitual state 6 months after the procedure, with no rest pain or wounds, but claudication at 500 m. Initially after the procedure the patient received dual antiplatelet therapy, but she was reinstated on apixaban from the cardiologist due to atrial flutter at dismissal from the hospital. She is currently on life-long anticoagulation and antiplatelet therapy with apixaban and ASA.

## Conclusions

While neointimal hyperplasia is a common occurrence, especially at the anastomotic site of vascular grafts, neointimal dissection is more rare. Iatrogenic dissections of the neointima has been reported from invasive cardiology procedures [2], as well as from prostetic arteriovenous fistulas [3]. A dissection can result from PTA or at the end of stents, but angiography alone may also pose a risk for dissection if the catheter tip is directed toward the wall of the graft and contrast media injected with force, for instance with the use of an automatic injector.

In our experience, the dissection can be mistaken for residual thrombus, and while continued thrombolysis will not solve this problem, the systemic effect of tPA can increase the risk of intracranial hemorrhage. As for intimal tears and subintimal dissections, it is important how the dissection flap influence hemodynamics. If there is no hemodynamic significant stenosis, and the tear does not pose risk to further progression to cause occlusion or thrombus formation, the dissection can be left untreated. If the flap needs treatment, prolonged balloon inflation can be attempted or a stent can be placed inside the graft.

In our experience, the neointima of stent grafts and grafts does not dissect easily, but when the dissection occur, it tends to extend rather long, often all the way to the anastomosis or the end of the stent graft. This is in contrary to iatrogenic subintimal dissections during vascular interventions, as they are often more limited, possibly due to atherosclerotic plaques and diseased vessel walls.

## Data Availability

Not applicable.

## Abbreviations

CTA: Computed tomography angiography
tPA: Tissue plasminogen activator
ASA: Acetylsalisylic acid
